# Array-CGH and multipoint FISH to decode complex chromosomal rearrangements

**DOI:** 10.1186/1471-2164-7-330

**Published:** 2006-12-29

**Authors:** Eva Darai-Ramqvist, Teresita Diaz de Ståhl, Agneta Sandlund, Kiran Mantripragada, George Klein, Jan Dumanski, Stefan Imreh, Maria Kost-Alimova

**Affiliations:** 1 Department of Microbiology, Tumour and Cell Biology (MTC), Karolinska Institutet, S-17177, Stockholm, Sweden; 2 Department of Pathology, Rudbeck Laboratory, Uppsala University Hospital, S-75185, Uppsala, Sweden; 3 Howell and Elizabeth Heflin Center for Human Genetics, University of Alabama at Birmingham (UAB), Medical School, Birmingham, AL 35294-0024, USA

## Abstract

**Background:**

Recently, several high-resolution methods of chromosome analysis have been developed. It is important to compare these methods and to select reliable combinations of techniques to analyze complex chromosomal rearrangements in tumours. In this study we have compared array-CGH (comparative genomic hybridization) and multipoint FISH (mpFISH) for their ability to characterize complex rearrangements on human chromosome 3 (chr3) in tumour cell lines. We have used 179 BAC/PAC clones covering chr3 with an approximately 1 Mb resolution to analyze nine carcinoma lines. Chr3 was chosen for analysis, because of its frequent rearrangements in human solid tumours.

**Results:**

The ploidy of the tumour cell lines ranged from near-diploid to near-pentaploid. Chr3 locus copy number was assessed by interphase and metaphase mpFISH. Totally 53 chr3 fragments were identified having copy numbers from 0 to 14. MpFISH results from the BAC/PAC clones and array-CGH gave mainly corresponding results. Each copy number change on the array profile could be related to a specific chromosome aberration detected by metaphase mpFISH. The analysis of the correlation between real copy number from mpFISH and the average normalized inter-locus fluorescence ratio (ANILFR) value detected by array-CGH demonstrated that copy number is a linear function of parameters that include the variable, ANILFR, and two constants, ploidy and background normalized fluorescence ratio.

**Conclusion:**

In most cases, the changes in copy number seen on array-CGH profiles reflected cumulative chromosome rearrangements. Most of them stemmed from unbalanced translocations. Although our chr3 BAC/PAC array could identify single copy number changes even in pentaploid cells, mpFISH provided a more accurate analysis in the dissection of complex karyotypes at high ploidy levels.

## Background

Genetic and epigenetic changes are among the hallmarks of cancer, with the illegitimate amplification of oncogenes and anti-apoptotic genes, and inactivation or deletion of tumour suppressors and pro-apoptotic genes as the main features. Positional cloning of such genes is dependent on high-resolution methods that can detect losses and gains of chromosome segments. Several techniques have been developed that can detect DNA copy number changes. Fluorescence *in situ *hybridization (FISH) was introduced in the early 1980s [1]. This is based on the use of chromosome region specific fluorescent-labelled DNA probes. It can dissect complex chromosomal aberrations, including very small sub-microscopic deletions and permits studies on interphase cells. It can reveal heterogeneity while also giving a general view of the whole karyotype. Large-scale analysis is time consuming, however, since it requires separate hybridizations and individual microscope analysis.

Comparative genomic hybridization (CGH) has facilitated the detection of DNA copy number changes. It was first restricted to metaphase chromosomes (metaphase CGH) [2]. However, recently high-resolution techniques have been developed using DNA fragments instead of metaphase chromosomes (array-CGH) [3]. The resolution of metaphase CGH is about 10 Mb, whereas array-CGH can resolve hundreds base pairs (oligo array-CGH) or hundreds kilo bases (BAC/PAC array-CGH) and permits the detection of cryptic copy number changes. Thus, the whole genome can be screened in a single experiment [3,4]. Array-CGH cannot detect balanced chromosomal translocations, insertions and inversions, however, and makes the analysis of heterogeneous and normal cell contaminated tumour samples difficult. Another drawback is that CGH only detects relative copy number changes and cannot be used to determine the real segmental copy number and overall ploidy [5]. In order to interpret ("decode") complex CGH profiles and to describe the underlying chromosome rearrangements, additional information is required. The array-CGH results may be validated by a reciprocal approach, FISH, that analyzes tumour cells with locus specific DNA.

In the present study, nine tumour cell lines were analyzed in order to establish rules that would facilitate the interpretation of array-CGH data. We focused our analysis on chr3 since it is frequently affected in solid tumours [6]. A 179 BAC/PAC based chr3 array-CGH was constructed, and the results of chr3 array analysis were compared with multipoint FISH (mpFISH), using the same 179 BAC/PAC clones as probes for interphase and metaphase chromosome analysis. On the basis of this analysis we propose a relatively simple approach to calculate locus specific copy number and ploidy. We show that the method can detect single copy number changes in up to near-pentaploid cells.

## Results

### Development of mpFISH and array-CGH for detailed chr3 analysis

**Figure 1:**
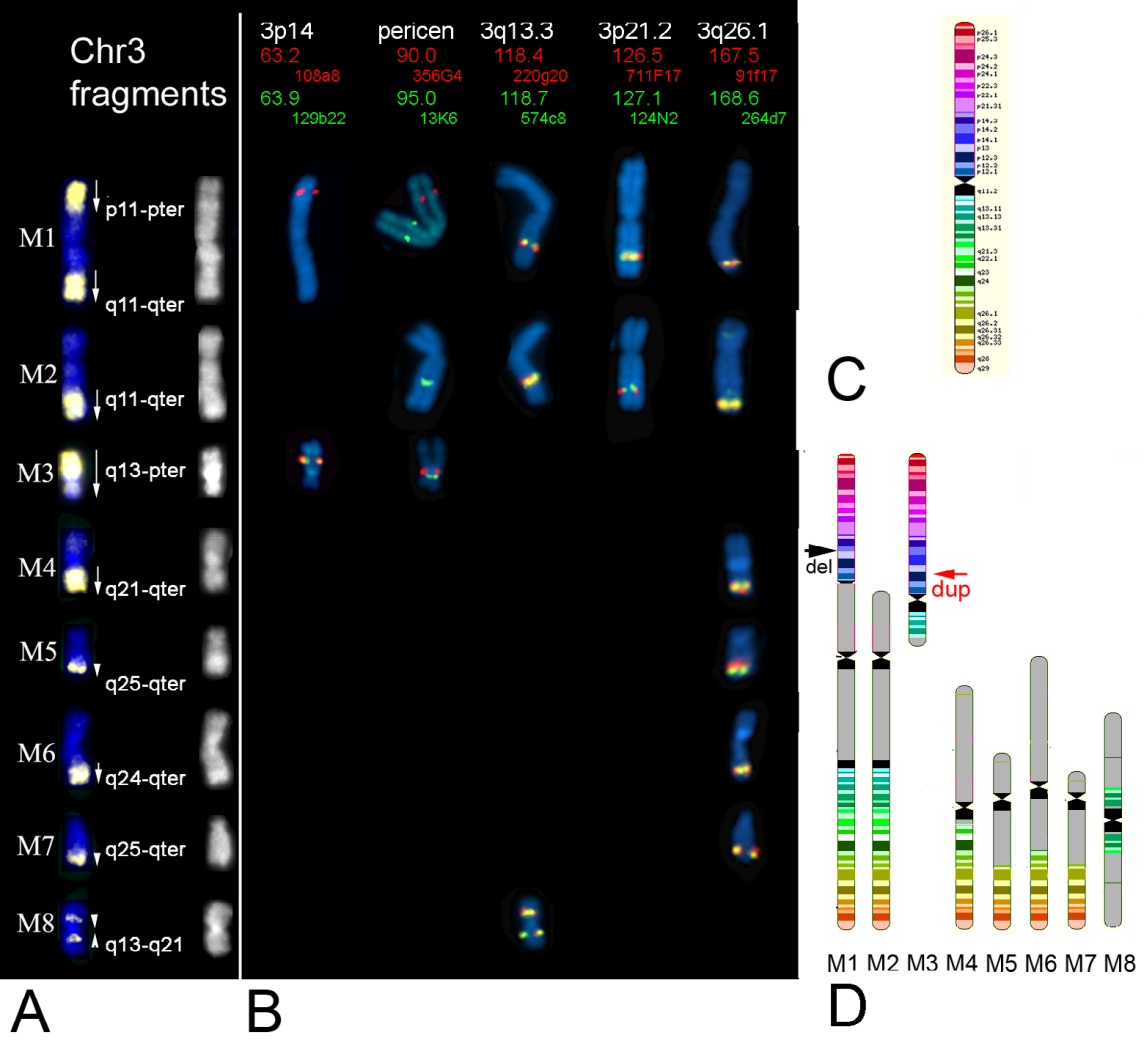
**Chr3 reconstitution in UOK147 based on chromosome painting and mpFISH**. **A**. Chr3 fragments, yellow, are identified by painting on rearranged marker chromosomes (M1, M2....M8). To the right DAPI banding. **B**. Examples of different mpFISH probe pair (red and green signals) localization on the rearranged chromosomes shown in A. White font on the top line: localization of the used pair on chr3 bands. Red and green fonts: the Mb position and the name of clone having red and green signals respectively. All the clones shown here belong to RP11 BAC library. **C**. Colour coding of banding pattern on the chr3 ideogram. **D**. Schematic colour coded representation of chr3 fragments in UOK147 on the rearranged marker chromosomes. Black arrow indicate a deletion site on M1, red arrow shows duplication on M3. Grey parts represent translocation partners from other chromosomes.

We have analyzed ten carcinoma cell lines with cytogenetic methods. The chromosome number was counted for a minimum 20 metaphase spreads in each cell line (Table 1 – Ploidy C). Subsequently, all lines were examined by chr3 painting. Several chr3 fragments were identified on differently rearranged marker chromosomes (M1, M2,...M8). All of them had a characteristic morphology that allowed their identification on metaphase plates (see Figure 1).

**Figure 2:**
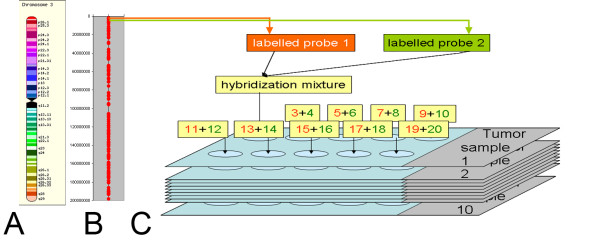
**Principle of mpFISH**. **A**. Colour coding of banding pattern on the chr3 ideogram. **B**. Distribution of 179 BAC/PAC sequences along the chr3 (from up to down) according to their base pair position. **C**. Scheme of mpFISH experimental set up. The BAC/PAC clone DNAs shown in B are labelled pair-wise with either biotin-dUTP(red) or digoxigenin-dUTP (green). 100–200 ng of biotin-labelled and the same amount of digoxygenin-labelled probe was dissolved in 10 μl of hybridization mixture and applied to corresponding area on each slide prepared from the ten different tumour cell line samples (1 μl of mixture containing 10–20 ng of each probe per area/slide). This procedure is repeated for all PAC/BAC probe pairs. Using this set up we were able to obtain FISH the results of 20 probes for ten samples in a single experiment. Hybridisation was carried out for 24–72 hours at 37°C under 9 × 9 mm cover slips sealed with rubber cement. Detection and microscopy are performed as described (see mpFISH technique description in Methods).

To identify the composition of each chr3 fragment on the marker chromosomes we developed a modified FISH method, called mpFISH. MpFISH can be introduced as a macro-array FISH, which is the reciprocal of micro-array CGH. While in array-CGH the BAC/PAC DNA is spotted in micro-dots on the slide and the tumour DNA is hybridized to it, in the mpFISH, the tumour nuclei and chromosomes are fixed and the different BAC/PAC DNAs are hybridized to multiple areas on the slide (usually 10), in pairs of two (or more) colour labelled probes (see Figure 2). By this improved FISH application, we reduced the amount of the probe to 100–200 ng of labelled probe DNA for the analysis of 10–15 samples. We could also analyze 20–24 probes in double-colour hybridisation on each slide in a single experiment. This has led to a considerable reduction in cost and time. A set of 179 commercially available, FISH mapped, chr3 specific BAC/PAC probes was used to analyze the ten cancer cell lines using interphase and metaphase mpFISH (see Methods). One cell line, as expected [7,8], had normal chrs3 and it was excluded from further analysis. In total 53 regions, which represent a row of neighbouring clones with the same copy number, were identified and characterized in the remaining nine cell lines (Table 1 – Regions). The majority of the chr3 rearrangements were generated by unbalanced translocations, but we could also identify interstitial deletions, duplications and even complex patterns of amplifications (Table 1 – Rearr FISH). The copy number of each region was calculated based on result of metaphase and interphase FISH (Table 1 – Copy nr FISH). Majority of the tumour cell lines, analyzed here, had almost homogeneous cell populations. In the cases of heterogeneous tumour cell lines, like TK164, the predominant population (>50%) copy number was used.

**Figure 3:**
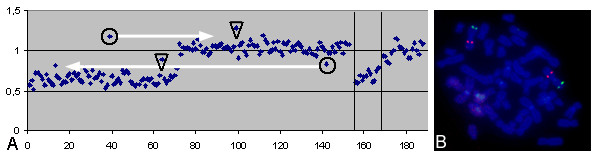
**Mismapped clones**. **A**. Array-CGH profile of UOK115 cell line. BAC/PACs covering chr3 are displayed in order on the X-axis from telomere of short arm (left), to telomere of long arm (right). Controls derived from chrX are plotted between the vertical lines right to chr3. Controls from autosomes are plotted to the right of the chrX controls. The Y-axis denotes the value of normalized fluorescence ratio (NFR) for each BAC/PAC clone (see Methods). The profile identifies 3p loss with ANILFR ± 1 sd; 0.7 ± 0.07. Two clones highlighted by circles, display outstanding fluorescence ratio than the neighbouring clones. On the plot arrows indicate real localizations revealed by FISH (RP11-129G16 locates at 3q12, clone RP11-732M7 at 3p24-25). Triangles indicate clones, which are part of segmental duplications identified by BLASTN. **B**. FISH image of normal metaphase with clones: RP11-994B16 (3q21.3), red, and the real localization of RP11-732M7, green, at 3q24-25.

**Figure 4:**
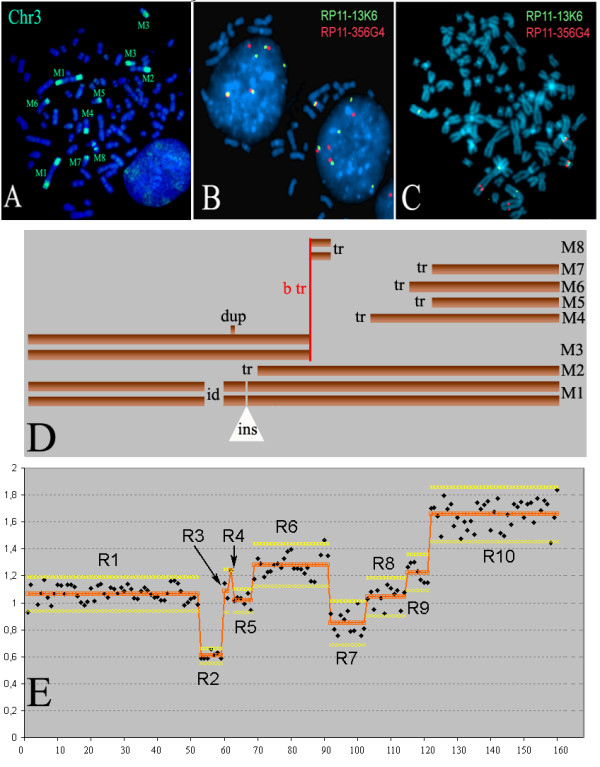
**Chr3 rearrangements in UOK147 cell line based on comparison of array-CGH and mpFISH results**. **A**. Chr3 painting probe identifies different chr3 fragments (green) on the rearranged marker chromosomes (M1, M2,...M8).**B **and **C**. Interphase (B) and metaphase (C) mpFISH illustration of a pericentromeric rearrangement. Clones RP11-356G4 (red) at 3p11, and RP11-13K6 (green) at 3q11, frame the centromere of chr3. Note the split signal indicating insertion in M1 and loss of red signal indicating loss of 3p through an imbalanced translocation in M2. **D**. Schematic results of chr3 rearrangements detected by mpFISH. Bars represent chr3 fragments found within the marker chromosomes (M1, M2,...M8). tr: unbalanced translocation breakpoint; b tr: balanced translocation; dup: duplication; id: interstitial deletion; ins: insertion of other chromosome fragment. **E**. Array-CGH profile. X-axis: displays 179 chr3 BAC clones ordered from 3pter (left) to 3qter (right). Y-axis: normalized fluorescence ratio (NFR). Each spot represents an average between at least two replicas of each clone on the array (see Methods). According to mpFISH we defined specific chr3 regions (R1, R2,...R10) of certain copy number: R1 ANILFR ± 1 sd; 1.07 ± 0.06 corresponds to 4 copies; R2 ANILFR ± 1 sd; 0.61 ± 0.03 corresponds to 2 copies a.s.o (see Table 1). Orange line represents the ANILFR (Average Normalized Inter-Locus Fluorescence Ratio) value and yellow lines frames the double standard deviation intervals for each region (Methods). (see Table 1). *(Note: the measurement points from individual PAC/BAC clones, which reside outside the double standard deviation, must be confirmed by FISH to be able to rely on them. In this case FISH confirmed only one single clone change -R4)*.

**Figure 5:**
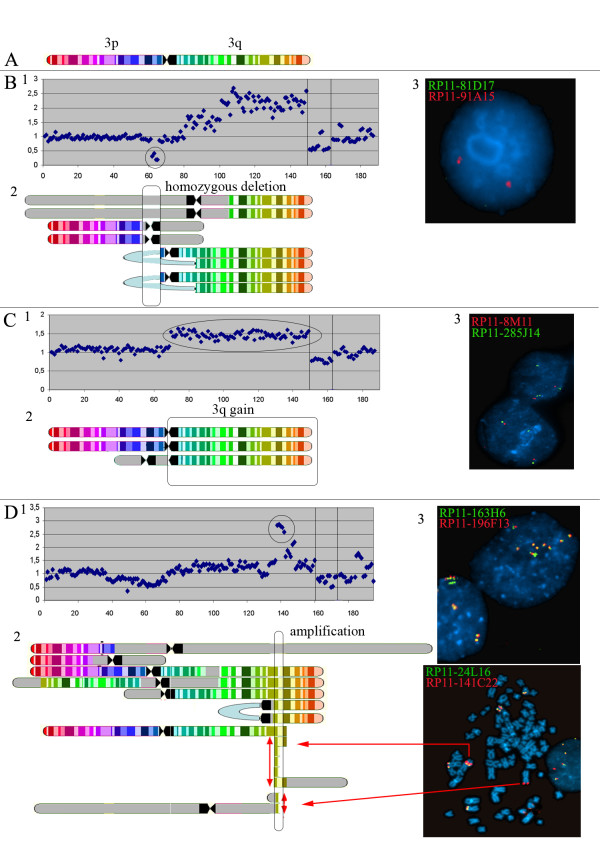
**Array-CGH profiles and mpFISH of three cancer cell lines displaying different chr3 aberrations**. **A**. Colour coding of banding pattern on the chr3 ideogram. **B**. Detection of homozygous deletion at 3p12-p13 in U2020. B1. Array-CGH detection of homozygous deletion (circle on the plot) (ANILFR ± 1 sd; 0.28 ± 0.1 as shown in Table 1). See for chart description legend to Fig 3. B2. Schematic representation of chr3 segments on the rearranged chromosomes according to mpFISH results. Grey parts represent translocation partners from other chromosomes. Blue loops show fusion between fragments. Rectangle box marks the chr3 region, corresponding to a homozygous deletion (0 copy number), missing in all rearranged chromosomes. Each piece from chr3 corresponds to the colour code given in A. B3. Interphase mpFISH visualizing the homozygous deletion. Probes RP11-91A15 (red) and RP11-81D17 (green) represent the region 3p11 outside and 3p12.2 within homozygous deletion, respectively. **C**. Terminal 3q gain in UOK125 using array-CGH (ANILFR ± 1 sd; 1.46 ± 0.08) (C1), metaphase mpFISH (C2) and interphase mpFISH (C3) (3 copies of 3q). The general layout follows the structure described for Fig 5B highlighting 3q gain of 3 copy number. On C3 RP11-8M11 (2 red signals on each nucleus) and RP11-285J14 (3 green signals on each nucleus) are located at 3p12.1 and 3q11.2 respectively. **D**. Amplification at 3q26 in HONE1 detected by array-CGH (ANILFR ± 1 sd; 2.81 ± 0.05) (D1), metaphase mpFISH (D2) and interphase mpFISH (D3) (13 copies). The general layout follows the structure described for Fig 5B. Arrows on D2 indicate duplication cycles within the amplified region. Interphase FISH image shows higher amplification of region at 173.25 Mb (RP11-163H6; green signals) compared to region at 173.65 Mb (RP11-196F13; red) within 3q26.31. On metaphase mpFISH image red signal corresponds to RP11-141C22 at 170.36 Mb, green signal corresponds to RP11-24L16 at 169.69 Mb within 3q26.2. Red arrows show amplified loci on rearranged chromosomes corresponding to our schematic representation on D2.

In parallel with the mpFISH analysis, a chr3 CGH array was set up covering both arms. It contained the same set of 179 chr3 BAC/PAC clones as used for the mpFISH experiments. Six chrX and 12 autosomal clones were used as controls. DOP-PCR amplified probes, generated by three different universal primers [9] were followed by aminolinker PCR, pooled and spotted on code-link slides. Array-CGH was performed on the same nine cancer cell lines. The ratios of the mean fluorescence intensity of test DNA versus reference DNA were calculated for each clone. The average ratio of the autosomal controls was used for normalization. Each dot on the array-CGH charts (Figures 3A, 4E and 5) represents the Normalised Fluorescent Ratio (NFR). The ANILFR (Average Normalized Inter Locus Fluorescent Ratio) and standard deviation (sd) values were calculated for each of the 53 mpFISH defined rearranged chr3 regions (see Methods) in order to assess the interlocus variation (Table 1 – ANILFR and sd).

### MpFISH identifies mismapped clones

Detailed analysis of array-CGH results is always required to exclude suspicious copy number changes. We could see several clones with extreme values in the majority of array-CGH profiles, which needed more detailed investigation. BAC/PAC clones were ordered on each array-CGH profile according to their Mb position on chr3 in the UCSC database [10]. By mpFISH, we verified the localization of the clones on normal chr3, and clones with wrong database localization could be readily identified. For example, clone RP11-129g16 has two locations in the UCSC database [10]: 54.3 – 54.5 Mb (3p14) and 102.2 Mb (3q12); mpFISH confirmed only the 3q12 localisation. This was in agreement with our array-CGH data where the copy number of this clone indicated that it was localized on 3q. MpFISH validated also the localization of RP11-732m7 at 3p24-25, while the UCSC database [10] placed it at 3q26 (170.9–171.1 Mb) (Figure 3).

There was a second group of clones that according to database information had well defined localisations on chr3, but they gave additional signals on other chromosomes upon mpFISH analysis and had extreme hybridization ratios on the array-CGH. These clones appeared to represent parts of segmental duplications detected by BLASTN (data not shown). These segmental duplications were not represented in the UCSC database [10], because the similarity was just below the database threshold (90% identity). The third category of clones was eliminated because they were localized on chromosomes other than chr3. Altogether 13 clones (7%) were eliminated from evaluation (Table 2).

### Chr3 aberrations by array-CGH and mpFISH

MpFISH analysis of the nine cell lines identified 53 regions of different copy number on chr3. Based on array-CGH results, we assessed the ANILFR and standard deviation for each region (Figure 4E and Table 1). The ANILFR values were different for regions with different copy numbers. Gains were found in 27 regions (bold in Table 1), losses in 13 (italics in Table 1). Losses were more frequent in 3p (10 out of 14 regions, 71%) while gains were more frequent on 3q (23 out of 26 regions, 88%).

We could visualize six interstitial losses (IL in Table 1). Only two were interstitial deletions (see region corresponding to R2 in Figure 4D,E); the other four were generated by two translocations (see region corresponding to R7 in Figure 4D,E). An example of interstitial loss is provided by the homozygous deletion (Figure 5B), previously characterized in a diploid small cell lung cancer cell line, U2020 [11-13]. On the array-CGH profile, the homozygous deletion is identified by clones that show a value of ANILFR ± 1 sd; 0.28 ± 0.1 (Figure 5B1). MpFISH using these probes could not detect any nuclear signal, due to a complete loss of this chr3 segment in U2020 (Figure 5B3). Reconstitution of different chr3 fragments on the marker chromosomes confirmed that the same region was lost (Figure 5B2). The analysis made also clear that the U2020 deletion was generated by three different translocations.

Only one of eight interstitial gains (IG in Table 1) detected on the array-CGH profiles was due to a simple duplication (see R4 in Figure 4D,E). The others were due to translocations (see R6 in Figure 4D,E).

All three terminal losses (TL in Table 1) and six terminal gains (TG in Table 1) originated from unbalanced translocations. An example of TG is shown on the array-CGH profile of UOK125 where the copy number of the whole 3q increases by one added copy, due to a translocation with breakpoint at the centromere (Figure 5C).

Sequential, stepwise changes were most frequent (16 regions, SC in Table 1). They were generated by intermediate copy number changes leading to interstitial or terminal gains/losses. They were initiated by translocations, followed by secondary rearrangements that generated further changes in copy number (see R9 and R10 in Figure 4E,D). Such stepwise changes have led to cumulative segmental amplifications in CAKI2 and HONE1 (Table 1). The array-CGH profile of copy number gain at 3q26 in HONE1 resulted from a complex rearrangement leading to the amplification of this segment (Figure 5D). Increase in copy number can be measured by interphase mpFISH and the rearrangements can be reconstituted by analyzing metaphase chromosomes in the same culture.

In addition to the rearrangements mentioned, we found three chr3 aberrations by mpFISH that were not detected by array-CGH: two insertions and one balanced translocation. Metaphase mpFISH on Figure 4A shows that the marker chromosome M1 in UOK147 had a chromosome fragment inserted in the pericentromeric region. This breakpoint was not identified by array-CGH (Figure 4E), because the copy number remained unchanged. In the case of properly selected probes, interphase mpFISH can detect insertion (split signals, Figure 4B). The change detected by array-CGH at this region was loss of the short arm of chr3 as a result of an unbalanced translocation in M2 (Figure 4).

It should be also mentioned that changes detected by array-CGH based on single clone data (see R4 on Figure 4E) should be always confirmed by additional methods, like FISH (see "dup" on Figure 4D).

### Correlation between ANILFR and segment copy number

**Figure 6:**
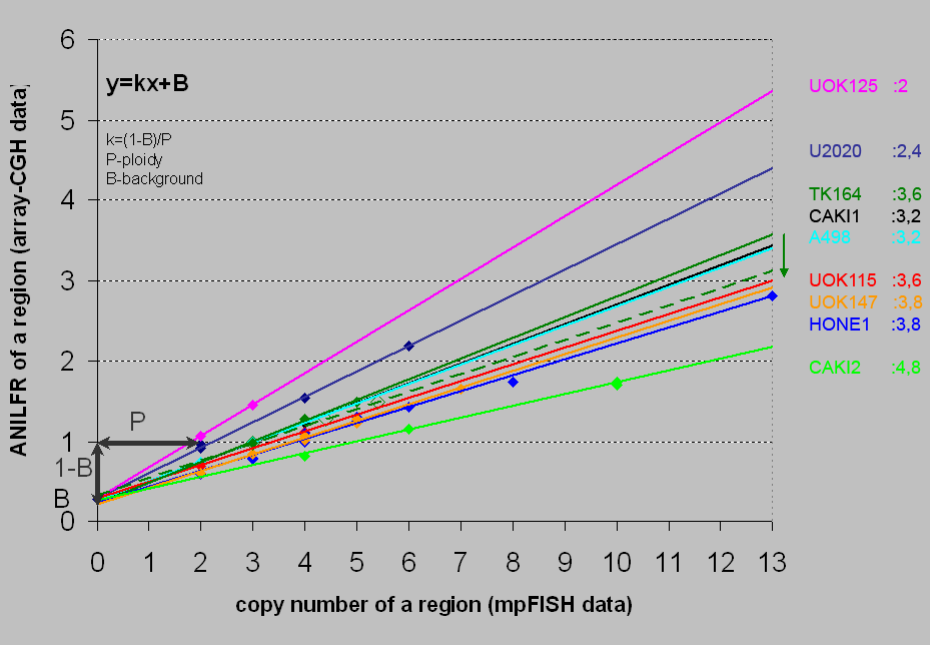
**Correlation between ANILFR (Average Normalized Inter-Locus Fluorescence Ratio) value identified by array-CGH and copy number determined by mpFISH**. X-axis: copy number of chr3 regions (R1, R2...) identified by interphase and/or metaphase mpFISH (see "Copy nr FISH" in table 1). Y-axis: ANILFR value for the same regions (see ANILFR in Table 1). As example for UOK125 cell line: Region R1, has an ANILFR of 1.07 (Y-axis value), which corresponds to 2 copies of this region on chr3 (X- axis value). (see Figure 5C and Table 1). These (x, y) coordinates define the first data point (pink romb) for the UOK125 trend line. The second data point corresponds to R2 with ANILFR of 1.46 (Y-axis value), and with a copy number of 3 (X- axis value). Each cell line has a number of data points (rombs of respective colour) corresponding to the number of chr3 region of different copy number (Table 1 – Region). Trend lines for each cell line were calculated and drawn. The interception of trend lines on Y-axis indicates the intensity of the background fluorescence "B" (corresponding to 0 copies, homozygous deletion region in U2020, ANILFR of 0.28). The slope of the trend lines reflects ploidy level. At the right from the graph, the ploidy number obtained from chromosome counts is shown for the respective cell lines ("Ploidy C" on Table 1). See other explanations in the text.

We compared ANILFR identified by array-CGH for regions that were determined by >3 clones, with copy number assessed by mpFISH (Table 1). Figure 6 shows the relationship between these two values. Each region with a certain copy number was represented by a point on the graph where the X-coordinate corresponded to the region copy number obtained from mpFISH analysis, while the Y-coordinate corresponded to its ANILFR. The number of data points was 2 in UOK125, UOK115; 3 in Caki1, A498 and TK164; 5 in U2020; 7 in Caki2; 10 in UOK147 and 11 in HONE1 equivalent to the number of regions with different copy numbers of the respective cell line. Based on these data points, the trend lines for each cell line (shown in different colours on Figure 6) were calculated in Excel. All nine trend lines followed the linear function of y = kx+B; where y is the ANILFR determined by array-CGH; x is the copy number from mpFISH. The constant B is equal to y = 0.28, corresponding to the crossing of all trend lines on y-axis. This value was equivalent also to ANILFR of the homozygous deletion in U2020, thus reflecting the background ANILFR. The other constant, k, reflects the slope of the trend line and is determined by the background ANILFR (B) and ploidy level (P) of the cell line: k = (1-B)/P (see black arrows on Figure 6). The fact that normalization was made according to the autosomal controls means that the value of x corresponding to y = 1 represents the average autosomal segment copy number, which corresponds to ploidy of the cell line (P). This formula shows that the copy number resolution of array-CGH (difference in ANILFR between two regions of consequent copy number) is inversely proportional to the ploidy level.

The expected copy number of a region (Table 1 – Copy nr E) was calculated using the formula y = kx+B, taking into account the ploidy from chromosome counts (Table 1 – Ploidy C) and the background ANILFR from homozygous deletion in U2020. These expected values corresponded well to the copy numbers determined by mpFISH (Table 1 – Copy nr FISH) with few exceptions (marked * in Table 1). The exceptional regions R2 and R3 in TK164 showed high levels of heterogeneity by mpFISH. Sixty percent of the cells had four R2 and five R3 copies. This was considered as a major abnormality and included in Table 1. However 40% of cells contained five R2 and six R3 copies. Taking this in consideration, we corrected the data points for this cell line, using as x-values, the average copy numbers between subpopulations (see dashed trend line in Figure 6). The exceptional amplified region R6 in CAKI2 showed also heterogeneity by FISH. The other exceptional regions were defined by too few (1–3) clones.

The expected ploidy was estimated (Table 1 – Ploidy E) as x-coordinate of trend lines at y = 1 (see P for UOK125 in Figure 6). A good correlation was found between these expected values and real ploidy based on chromosome counts.

### Copy number resolution of array-CGH

For 52 out of 53 regions ANILFR ± 1 sd value was different from adjacent ones, (see Table 1), showing that copy number change was detected by array-CGH in up to pentaploid cells. In attempts to generalise the results, we calculated the expected ANILFR difference for single copy number change using y = kx+B formula. This difference in cell lines of ploidy 2, 3, 4 and 5 would be 0.36; 0.24; 0.18 and 0.15 respectively. Median sd for all regions was 0.07. The difference between the double sd (0.14) of two regions allowed a distinction between these regions. More than 0.14 differences characterised single copy number change in all cells irrespective of ploidy, making it possible to identify single copy number changes in array-CGH. Taking into account the fact that the vast majority (43 out of 47) of the sd values lied in a range up to 0.12, we concluded that single copy number change can be determined highly efficiently in di- and triploid cells and less efficiently in tetra and pentaploid cells.

The vast majority of ANILFR values were fitting in the +/- 2sd interval (see Figure 4E). Therefore the precise location of the breakpoint can be made generally at the transition, where ANILFR changes more than 4x median sd (0.3). Such difference is characteristic for single copy number changes in diploid cell lines. For tri- tetra- and pentaploid cells, it would be difficult to identify breakpoints of single copy number transitions (see transition R3-R4; R4-R5; R5-R6; R7-R8 and R8-R9 for UOK147 in Figure 4E). The difference of more than 0.3 characterised double copy number changes, therefore breakpoints could be identified with high confidence for 2-copy transitions (see transition R1-R2; R2-R3; R6-R7 and R9-R10 for UOK147 in Figure 4E).

## Discussion

Most solid tumours have complex karyotypes with several chromosomal rearrangements. The combination of high-resolution methods can describe these changes efficiently. In this study, we have chosen nine cell lines with complex karyotypes to evaluate the effectiveness of different methods. Array-CGH, interphase and metaphase mpFISH gave corresponding results. In the case of complex rearrangements not followed by copy number changes (insertions, inversions and reciprocal translocations); array-CGH and interphase mpFISH could not detect the rearrangements in contrast with metaphase mpFISH that did. However one drawback is that metaphase mpFISH requires high mitotic frequency in the analyzed cell population, which limits its application for tumour biopsy analysis. Thus combining metaphase, interphase mpFISH and array-CGH permitted the detailed characterization of the reorganized chr3 segments.

The losses and especially gains seen on array-CGH profiles reflected complicated and clearly sequential chromosome rearrangements. This is shown by the high incidence of cumulative changes in our analysis corresponding to sequential unbalanced translocations, leading to the accumulation of fragments carrying a gain-region, often in the terminal part of 3q. Even high copy number amplifications detected in HONE1 and CAKI2 were the consequences of cumulative changes of different types (duplications, unbalanced translocations, isochromosome formation). Each of these stepwise changes should provide the cell with selective growth advantage, supporting the importance of single copy number changes in cancer development.

Array-CGH may have its limitations in determining ploidy and it is controversial whether it can determine the exact copy number of different chromosome regions [4]. Some publications claim a linear relation between the theoretical and measured copy number changes [14]. In our present study we set on to analyse the relationship between copy number and ANILFR for several regions in cell lines of different ploidy. Based on a comparison of seven cell lines having >2 data points, we found strong support for a linear relation (see Figure 6). We have also shown that the trend line functions can be described by an equation that permits the estimation of the copy number of a certain chromosome region, which corresponded to our experimental results. The actual copy number is the function of parameters that include the variable ANILFR, and two constants, the ploidy and the background. Background ANILFR can vary depending on the array-CGH platform [14] and experimental conditions in a particular lab. It can be determined by using a cell line (ex.U2020) containing a large homozygous deletion. In order to estimate the real copy number for each ANILFR, it would thus be important to know the cell ploidy. If this is missing, at least one locus copy number has to be determined by FISH.

It may be concluded from the proposed formula (y = kx+B, where y is the ANILFR; x is mpFISH copy number; B is background ANILFR; k = (1-B)/P is ploidy), that the array-CGH results would be mainly influenced by cell ploidy under certain conditions. We estimated the copy number resolution of our PAC/BAC based array-CGH and showed that single copy number changes can be detected even in pentaploid cells, although the most reliable results are obtained in di- and triploid samples. However it may be more difficult to identify a breakpoint position since it can be mapped precisely only in diploid cells.

The heterogeneity and the normal cell contamination of tumours need to be considered as well. Heterogeneity and the presence of subpopulations occur frequently even in cell lines. The level of heterogeneity can influence the interpretation of copy number changes in array-CGH as we have shown for the TK164 cell line. In contrast, mpFISH determines the copy number at single cell level and thus allows identification of subpopulations. Normal cell contamination needs to be considered as well. It is useful to use log2 graphical representations of array-CGH that visualize small differences in fluorescence signal ratios more accurately. MpFISH counts on single cell level provide the most accurate analysis.

## Conclusion

We have evaluated the effectiveness of array-CGH and mpFISH in characterizing chr3 rearrangements in nine karyotypically complex cell lines. We have found the following:

1. The losses and gains seen on array-CGH profiles are projections of complex sequential chromosome rearrangements leading to the loss or accumulation of specific fragments.

2. The analysis of the correlation between real copy number from mpFISH and the ANILFR value detected by array-CGH showed that the copy number is a linear function of parameters that include the variable, ANILFR, and two constants, ploidy and background normalized fluorescence ratio.

3. With our 1 Mb PAC/BAC based chr3 array-CGH, a single copy number change can be detected even in pentaploid cells, but the most reliable results are obtained in di- and triploid samples. It is more difficult to identify the breakpoint position which can only be mapped precisely in diploid cells.

4. In heterogeneous or normal cell contaminated samples the most accurate analysis can be made by mpFISH.

## Methods

### Cell lines

In our study nine cancer cell lines were analyzed. They were selected according to their ploidy level and chr3 rearrangements determined by chr3 painting and metaphase chromosome counts. Seven renal cell carcinoma cell lines: A498, UOK115, UOK125, UOK147, TK164 [15-18], CAKI1 (ATCC catalog No.HTB46) and CAKI2 (ATCC catalog No.HTB47); one small cell lung cancer cell line U2020 [11-13] with an interstitial homozygous deletion on 3p12.3 and a human nasopharyngeal carcinoma cell line HONE1 [19-23] were used. All cell lines were cultured on IMDM medium with 10% fetal calf serum, 1% penicillin-streptomycin and 1% Glutamine. For the array experiments total genomic DNA was isolated using GeneElute mammalian genomic DNA miniprep kit (Sigma-Aldrich, Germany). For the mpFISH experiments cells were treated with 20 μg/ml colcemid for 3–4 h to obtain metaphase chromosomes. After treatment with hypotonic solution cells were fixed in methanol: acetic acid (3:1) following standard protocols.

### BAC/PAC clones

A set of 179 BAC/PAC clones was selected according to several considerations: (i) DNA from all these clones was commercially available (2 × 96 deep well blocks) from BACPAC Resources Center, CHILDREN'S HOSPITAL OAKLAND, Oakland, USA [24]; (ii) all clones were FISH mapped, at least partially sequenced and their localization was approved using UCSC database [10]; (iii) all clones were chr3 specific and covered the whole chromosome with a resolution of ~1 Mb.

### ArrayCGH

DNA labelling, hybridization and post-hybridization processing, scanning and image analysis were performed as previously described [16]. A pool of peripheral-blood-derived DNA from 10 normal females was used as normal reference DNA for all hybridizations performed. In brief, 2 μg of test DNA and 2 μg of reference DNA were differentially labelled by random priming using Cy3-dCTP (PA53021, GE Healthcare, Piscataway, NJ) and Cy5-dCTP (PA55021, GE Healthcare). These were then mixed with 100 μg of Cot-1 DNA (Roche, Basel, Switzerland) and hybridized to the array. Image acquisition was performed using the GenePix 4000B scanner (Axon Instruments Inc., Union City, CA). Analysis of spot intensities was carried out using GenePixPro image analysis software (Axon Instruments). The average and coefficient of variation of fluorescence ratios for each measurement point were calculated. Data points displaying a coefficient of variation greater than 5% between at least two of the replica spots were discarded from further analysis. The average of fluorescence ratios from autosomal controls was used in the normalization of data in each hybridization experiment. The ANILFR (Average Normalized Inter Locus Fluorescent Ratio) values were calculated in order to assess the inter-locus variation, representing region(s) on the array. The NFR (Normalized Fluorescence Ratio) for successfully scored loci from a certain, continuous region on the array, which corresponded to mpFISH identified regions, was used to calculate the ANILFR value and standard deviation.

### mpFISH

Metaphase chromosomes and interphase nuclei were used from methanol:acetic acid (1:3) fixed cells. We analysed a minimum of 20 metaphases in each sample using FISH with chr3 specific painting probe labelled with FITC (Cambio, Cambridge, UK). In each metaphase, we identified all labelled normal and marker chromosomes.

Using multi-channel pipette 5 μl of fixed cell suspension was applied to the slide to obtain 10 hybridization fields, marked on the back-side of the slide. The slides were pre-treated with pepsin and prefixed before denaturation. As probes, 200 ng of commercial BAC/PAC DNA (see BAC/PAC clones) was labelled with nick-translation either with biotin-dUTP or digoxigenin-dUTP (Nick Translation Mix; Roche Molecular Biochemicals, Mannheim, Germany). Two-colour mpFISH in microvolumes (1 μl) was performed on 10 sites/slide under 9 × 9 mm cover slips. The hybridization technique was used as described previously [25]. The biotin labelled probes was detected with Cy3 conjugated streptavidin (Amersham Biosciences, GE Healthcare Worldwide) and digoxigenin labelled probes with FITC conjugated anti-digoxigenin antibodies (Roche Molecular Biochemicals Mannheim). Between 100–200 interphase nuclei and 10–20 metaphase plates were analysed for each sample using a fluorescence microscope (Leitz-DMRB, Leica, Heidelberg, Germany) equipped with a Hamamatsu C 4800 cooled CCD camera (Hamamatsu, Herrsching, Germany) and Adobe Photoshop 7.0 (Adobe Systems, San Jose, Calif., USA).

## Authors' contributions

EDR contributed to the designed the study, carried out the cell culture, cytogenetical and molecular studies, database searches and drafted the manuscript. TDS helped with the array-CGH experiments and drafting the manuscript, AS helped with cytogenetical studies, KM printed the chr3 array. GK, JD and SI participated in coordination, discussions related to result interpretation and drafting of the manuscript. MKA have made substantial contribution to conception and design of the study, to result interpretations and have been actively involved in the drafting the manuscript. All authors read and approved the final manuscript.

## Figures and Tables

**Table 1:** Chr3 rearrangements based array-CGH data and mpFISH counts

**Nr**	**Cell Line**	**Ploidy C**	**Ploidy E**	**Region**	**Rearr FISH**	**Copy nr FISH**	**Copy nr E**	**ANILFR**	**sd**	**Abn CGH **
1	**U2020**	2.4	2.2	R1		2	2.29	0.97	0.06	No
2				R2	tr tr	0	0.01	*0.28*	0.1	IL
3				R3		2	2.14	0.92	0.1	no
4				R4	tr	4	4.18	**1.54**	0.18	SC
5				R5	tr	6	6.32	**2.19**	0.23	TG
6	**UOK125**	2	1.8	R1		2	2.19	1.07	0.07	no
7				R2	tr	3	3.26	**1.46**	0.08	TG
8	**CAKI1**	3.2	3	R1	tr	2	2.06	*0.74*	0.05	TL
9				R2		3	3.10	0.98	0.05	no
10				R3	tr	4	4.21	**1.23**	0.05	TG
11	**A498**	3.2	3	R1		3	3.13	0.98	0.06	no
12				R2	tr tr	2	2.08	*0.75*	0.07	IL
13				R3		3	3.15	0.99	0.06	no
14	**TK164**	3.6	3	R1		3	3.50	0.98	0.05	no
15				R2	tr	4	4.97*	**1.28**	0.08	SC
16				R3	tr	5	6.03*	**1.49**	0.08	TG
17	**UOK115**	3.6	3.4	R1	tr	2	1.88	*0.7*	0.07	TL
18				R2	tr	4	3.71	**1.12**	0.06	TG
19	**HONE1**	3.8	3.8	R1		4	4.02	1.04	0.1	no
20				R2	tr	3	2.73	*0.79*	0.06	SC
21				R3	id id	1	0.34*	*0.34*	nc	IL
22				R4	tr	3	2.69	*0.79*	0.06	SC
23				R5	tr tr	2	1.72	*0.6*	0.06	IL
24				R6	tr	3	2.63*	*0.78*	0.08	SC
25				R7		4	4.37	1.11	0.1	no
26				R8	tr tr	5	5.15	**1.26**	0.11	IG
27				R9		4	3.80	1	0.05	no
28				R10	ampl	6	6.04	**1.43**	0.04	SC
29				R11	ampl	13	13.22	**2.81**	0.05	IG
30				R12	ampl	10	11.96*	**2.57**	nc	SC
31				R13	ampl	8	7.65	**1.74**	0.12	SC
32				R14	ampl	5	5.41	**1.31**	0.12	SC
33	**UOK147**	3.8	3.8	R1		4	4.14	1.07	0.06	no
34				R2	id	2	1.75	*0.61*	0.03	IL
35				R3		4	4.26	1.09	0.08	no
36				R4	du	5	5.04	**1.24**	nc	IG
37				R5		4	3.90	1.02	0.04	no
38				R6	tr tr	5	5.26	**1.28**	0.08	IG
39				R7	tr tr	3	3.02	*0.85*	0.08	IL
40				R8		4	4.03	1.04	0.07	no
41				R9	tr	5	4.96	**1.22**	0.07	SC
42				R10	tr	7	7.22	**1.66**	0.1	TG
43	**CAKI2**	4.8	5	R1	tr	4	3.58	*0.81*	0.06	TL
44				R2	ampl	6	5.83	**1.16**	0.08	SC
45				R3	ampl	10	9.67	**1.74**	0.02	SC
46				R4	ampl	14	14.98*	**2.55**	0.01	IG
47				R5	ampl	6	5.53	1.11	nc	no
48				R6	ampl	10	9.39*	**1.7**	0.1	IG
49				R7	ampl	6	5.57*	**1.12**	nc	SC
50				R8	ampl	10	8.78*	**1.61**	0.2	IG
51				R9	ampl	6	5.82	**1.15**	0.25	SC
52				R10	ampl	14	13.31*	**2.29**	nc	IG
53				R11	ampl	6	5.79	**1.15**	0.08	SC

"Ploidy C": ploidy determined from chromosome counting in relation to haploid number. "Ploidy E": expected ploidy calculated based on trend lines in Figure 6 (see details in the text). "Region": continuous chr3 parts containing a row of neighbouring clones (R1, R2...) of different copy number in the respective cell lines (see e.g. regions R1, R2... R10 in UOK 147 on Fig 4E). "Rearr FISH": chromosome rearrangements identified by metaphase mpFISH leading to copy number change (tr- translocation; id- interstitial deletion; du- duplication; ampl- amplification). "Copy nr FISH": copy number of the region identified in mpFISH experiments. "Copy nr E": expected copy number of the region counted by y = kx+B formula (*- exceptions, where the expected copy number was different from the real copy number due to heterogeneity or few data points). "ANILFR": average normalized inter-locus fluorescence ratio of the region; italics: lost regions and bold: regions of gain. "sd": standard deviation between NFR values within the region. "Abn CGH": chr3 abnormality detected by array-CGH. (no- normal; IL- interstitial loss; IG- interstitial gain; SC- step-wise change; TG- terminal gain; TL- terminal loss).

**Table 2:** List of eliminated clones based on their false positive copy number change found by array CGH in the majority of analyzed cell lines

**Clone name**	**Comment**
Rp11-91k4	Segmental duplication identified by BLASTN
Rp11-208g16	Segmental duplication identified by BLASTN
Rp11-119l2	Segmental duplication identified by BLASTN
RP11-168f7	Localization on other chromosome (chr19)
CDT-2278f18	Segmental duplication identified by BLASTN
RP11-129g16	Misplacement in database; real localization on 3q
RP11-79o5	Segmental duplication identified by BLASTN
RP11-666d14	Not functioning probe in any experiment
Rp11-153k19	Probe localized on other chromosome
Rp11-994b16	Segmental duplication identified by BLASTN
Rp11-732m7	Misplacement in database; real localization on 3p
Rp11-80p4	Localization on other chromosome (chr4)
Rp11-125e8	Segmental duplication identified by BLASTN
